# The Late-Gestational Triglyceride to High-Density Lipoprotein Cholesterol Ratio Is Associated with Neonatal Macrosomia in Women without Diabetes Mellitus

**DOI:** 10.1155/2020/7250287

**Published:** 2020-06-19

**Authors:** Mengru Yu, Wenting Wang, Hong Wang

**Affiliations:** ^1^Center for Reproductive Medicine, Cheeloo College of Medicine, Shandong University, Jinan, Shandong, China; ^2^Key laboratory of Reproductive Endocrinology of Ministry of Education, Shandong University, Jinan, Shandong, China; ^3^Shandong Key Laboratory of Reproductive Medicine, Jinan, Shandong, China; ^4^Shandong Provincial Clinical Research Center for Reproductive Health, Jinan, Shandong, China; ^5^National Research Center for Assisted Reproductive Technology and Reproductive Genetics, Shandong University, Jinan, Shandong, China; ^6^Department of Obstetrics and Gynecology, The Second Hospital of Shandong University, Jinan, China; ^7^Department of Obstetrics and Gynecology, Shandong Provincial Hospital Affiliated to Shandong University, Jinan, China

## Abstract

**Objective:**

To investigate the associations between late-gestational dyslipidemia, expressed as the ratio between triglycerides (TGs) and high-density lipoprotein cholesterol (HDL), and the risk of macrosomia among nondiabetic pregnant women.

**Methods:**

In this case-control study, 171 pregnant women who delivered macrosomia newborns were recruited from a total of 1856 nondiabetic pregnant women who delivered a singleton, nonanomalous newborn. A total of 684 normal controls were one-to-four matched by age. Logistic regression analysis was used to analyze the association between the TG/HDL ratio and the neonatal body weight as well as the risk of macrosomia.

**Results:**

The maternal serum TG and TG/HDL levels were much higher in the macrosomia group, while the maternal serum HDL-C levels were much lower in the macrosomia group than those in the control group. However, the serum total cholesterol (TC) and LDL-C levels were not significantly different between the two groups. Furthermore, maternal TG/HDL levels were positively associated with neonatal body weight. The confounding factors including maternal age, maternal height, gestational age, maternal body mass index (BMI), FPG, SBP, and neonatal sex were adjusted. A positive association between TG/HDL and neonatal body weight was still found. Moreover, the prevalence of macrosomia increased markedly in a dose-dependent manner as with maternal TG/HDL levels increased.

**Conclusions:**

Maternal serum TG/HDL levels at late gestation are positively associated with neonatal body weight and the risk of macrosomia in women without DM. Maintaining maternal lipid levels in an appropriate range is important in the context of fetal overgrowth and primary prevention of macrosomia.

## 1. Introduction

Macrosomia is defined as a neonatal birth weight greater than or equal to 4000 g [1], which is a risk factor for adverse maternal and fetal perinatal outcomes [2]. Mothers of newborns with macrosomia are at an increased risk of prolonged labor, labor augmentation with oxytocin, cesarean delivery, abnormal hemorrhage, infection, and perineal trauma [3], while macrosomic newborns have a greater risk for perinatal asphyxia, meconium aspiration, shoulder dystocia, and death [2, 4, 5]. Furthermore, macrosomic newborns are at an increased risk of metabolic disorders such as type 2 diabetes mellitus (T2DM), obesity, and hypertension in adulthood [6–8].

An increasing prevalence of macrosomia has been reported in both developed countries and developing countries. Macrosomia prevalence in developed countries is between 5% and 20% [4]. In China, it was reported that the prevalence of macrosomia increased from 6.0% in 1994 to 7.8% in 2005 [9]. A variety of factors have been reported to be associated with an increased risk of macrosomia, such as gestational diabetes mellitus (GDM), older maternal age, excessive gestational weight gain, and male fetal sex [10–12], and the prevalence of diabetes and obesity in women of reproductive age has been thought to be more important factors [13, 14].

Maternal lipids play an important role in fetal development [15], and mildly elevated triglycerides (TGs) and cholesterol are detected throughout pregnancy [16], while abnormal elevation of lipids during pregnancy, especially in the GDM population, has been associated with adverse pregnancy outcomes, including gestational hypertension, large-for-gestational-age (LGA) newborns, and preterm birth (PTB) [17–19]; the effects of maternal dyslipidemia on pregnancy outcomes in the nondiabetic population are inconsistent. Some studies have found that newborn birth weight and the risk of macrosomia or LGA are positively correlated with blood TGs or total cholesterol (TC) [20–22] and negatively correlated with high-density lipoprotein cholesterol (HDL-C) [23, 24], while some other studies found no significant correlation between newborn birth weight and blood lipids [25, 26].

The ratio of TGs to HDL is a commonly used marker for lipid disturbance. TG/HDL has been established as a surrogate marker for insulin resistance [27] and endothelial dysfunction [28]. One study reported that a pregestational TG/HDL ratio ≥3 up to 1 year before pregnancy was associated with an increase in adverse pregnancy outcomes, mainly GDM and preeclampsia [29]. However, the association of TG/HDL in late gestation with macrosomia, especially in the nondiabetic population, is still unknown.

In the present study, we evaluated the association between the maternal TG/HDL ratio in late gestation and the risk of macrosomia among a large group of Chinese women without DM.

## 2. Methods

### 2.1. Study Design and Groups

The present retrospective case-control study included all women who were admitted to Shandong Provincial Hospital affiliated to Shandong University and delivered between January 2013 and December 2014. This study was approved by the Ethics Committee of Shandong Provincial Hospital affiliated to Shandong University, and written informed consent was obtained from every participant before enrollment. The study was performed in compliance with the Declaration of Helsinki.

The inclusion criteria were as follows: (1) live-born singleton pregnancy; (2) delivery at 37–42 gestational weeks; (3) natural conception; and (4) integrated medical records and a clear gestational age. The exclusion criteria were as follows: (1) pregestational DM or GDM history; (2) thyroid function disorder; (3) liver function disorders (ALT or AST > 2.5‐fold the upper limit), renal diseases (serum creatinine ≥ 105 *μ*mol/L); (4) malignant diseases, inflammatory bowel diseases, and documented malabsorption; (5) delivery before 37 weeks; (6) fetal congenital malformation, multifetal gestation or birth weight less than 2500 g; and (7) incomplete data.

GDM was diagnosed according to the IADPSG criteria 2 h OGTT: fasting ≥ 92 mg/dL (5.1 mmol/L), 1 h > 180 mg/dL (10.0 mmol/L), and 2 h ≥ 153 mg/dL (8.5 mmol/L) [30].

In this study, the sample-size calculation was performed by the two-sided two-sample *t*-test module of PASS 2008 software, in which *α* = 0.05 (2-sided) and *β* = 0.10.

**Figure 1:**
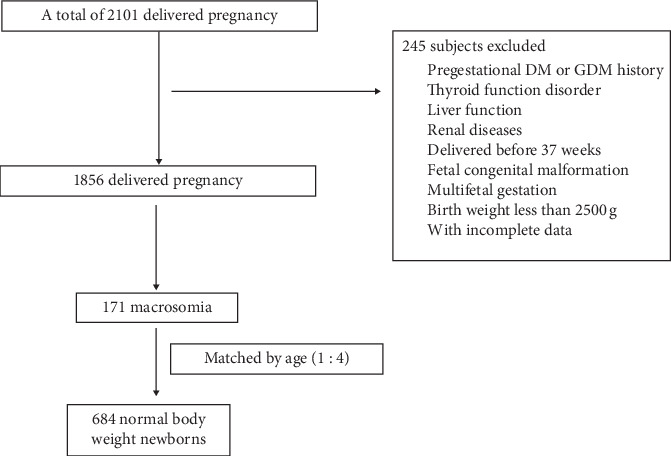
The flowchart of the analyzed population selection.

Finally, 171 pregnant women who delivered macrosomia newborns were recruited from a total of 1856 nondiabetic pregnant women, who delivered a singleton, nonanomalous newborn, and 684 pregnant women who delivered normal body weight newborns were individually matched at a one-to-four ratio with the cases by age (Figure 1).

### 2.2. Data Collection

All the data were extracted from medical records, including maternal age, height, weight before delivery, blood pressure, DM/GDM history, gestational age, gravidity, mode of delivery, neonatal birth weight, birth length, and neonatal sex. Height was determined to the nearest 1 cm, and weight was determined to the nearest 0.5 kg. Body mass index (BMI) was calculated as the weight in kilograms divided by the square of the height in meters. Blood samples were drawn between 37 and 42 weeks of gestation after overnight fasting. Biomedical analyses, including fasting plasma glucose (FPG), serum TG, total cholesterol (TC), high-density lipoprotein cholesterol (HDL-C), low-density lipoprotein cholesterol (LDL-C), and serum uric acid (UA), were performed in the clinical laboratory center of Shandong Provincial Hospital affiliated to Shandong University.

### 2.3. Statistical Analysis

All statistical analyses were performed using SPSS software (25.0 for Windows, SPSS Inc., Chicago, USA). Values are presented as the mean ± standard deviation, median (interquartile range), or number (percentage).

Differences between groups were tested using the independent two-sample *t*-test, the Mann–Whitney test, and the chi-squared test. Pearson's correlation analysis was performed between neonatal body weight and clinical characteristics. A multivariate linear regression analysis was performed with neonatal body weight as the dependent variable and other variables as independent variables to identify independent factors affecting neonatal body weight. All statistical tests were two-tailed, and statistical significance was defined as *P* < 0.05.

## 3. Results

### 3.1. Characteristics of the Study Population

The basic maternal and neonatal clinical characteristics of the study population are shown in Table 1. Regarding maternal characteristics, the maternal height (164.84 ± 4.51 vs. 163.16 ± 4.85, *P* ≤ 0.001), weight (80.24 ± 10.54 vs. 72.77 ± 8.98, *P* ≤ 0.001), and BMI (29.52 ± 3.76 vs. 27.33 ± 3.24, *P* ≤ 0.001) were higher in the macrosomia group than in the normal birth weight group. The SBP (121.46 ± 11.37 vs. 118.98 ± 11.46, *P*=0.012) and DBP (77.81 ± 8.71 vs. 76.11 ± 9.14, *P*=0.025) levels were much higher in the macrosomia group than in the normal birth weight group. However, the prevalence of hypertension was not different between the two groups. In addition, maternal age, FPG, UA, creatine, AST, and ALT showed no significant differences between the two groups.

For the neonatal characteristics, compared with the normal birth weight group, the macrosomia group included more male neonates (59.65 vs. 50.73%, *P*=0.037), the gestational age was higher (39.99 ± 0.91 vs. 39.67 ± 1. 08, *P* < 0.001), and the neonates were longer (51.63 ± 1.45 vs. 49.33 ± 1.77, *P* < 0.001). Therefore, cesarean delivery was more frequent (56.14% vs. 45.76%, *P*=0.015) in women delivering macrosomic neonates than in women with normal birth weight pregnancies. There was no difference in gravidity between the two groups.

### 3.2. Lipid Profile of the Study Population

As shown in Table 2, the serum TC and LDL-C levels were not significantly different between the two groups. However, the serum TG levels in the macrosomia group were much higher than those in the normal birth weight group (3.64 mmol/L vs. 2.57 mmol/L, *P* < 0.001), while the serum HDL-C levels were much lower in the macrosomia group (1.80 ± 0.38 vs. 2.15 ± 0.51 56, *P* < 0.001) than those in the normal birth weight group.

We also used the ratios of different serum lipids to evaluate the lipid profile change between the two groups. As shown in Table 2, TG/HDL and TG/LDL were much higher in the macrosomia group than in the normal birth weight group (2.27 ± 1.22 vs. 1.26 ± 0.43, *P* < 0.001; 1.29 ± 0.93 vs. 0.80 ± 0.35, *P* < 0.001, respectively). LDL/HDL was also higher in the macrosomia group than in the normal birth weight group (1.90 ± 0.60 vs. 1.68 ± 0.55, *P* < 0.001).

### 3.3. TG/HDL Was Positively Associated with Neonatal Body Weights

Spearman's correlation analysis was performed on neonatal body weights and clinical characteristics. As shown in Table 3, for the correlation between neonatal body weight and maternal lipid profile, we found that neonatal body weight was positively associated with serum TG levels, TG/HDL, and LDL/HDL but had no association with serum TC and LDL-C levels. In addition, neonatal body weight was positively associated with gestational age, maternal BMI, maternal FPG levels, and birth length.

Furthermore, a multivariate linear regression analysis was performed to study the association between TG/HDL and neonatal body weight. As shown in Table 4, TG/HDL was positively associated with neonatal body weight. When we adjusted for the effect of maternal BMI and FPG, TG/HDL was still positively associated with neonatal body weight. We adjusted more factors that have been reported to have an effect on neonatal body weight, including maternal age, gestational age, maternal height, BMI, FPG, SBP, and neonatal sex, and a positive association between TG/HDL and neonatal body weights was still found. The goodness of fit of our multivariate linear regression model was 0.545.

### 3.4. Maternal TG/HDL Levels Are Associated with Macrosomia Risk

We stratified the entire study population into 4 groups (quartiles) according to the TG/HDL levels to examine the extent of macrosomia risk across the spectrum of TG/HDL levels. As serum TG/HDL increased, the risk for macrosomia gradually increased after adjustment for potential confounding factors. As shown in Table 5, compared with the group with serum TG/HDL levels lower than 1.02, the risk for macrosomia markedly increased by approximately 13-fold when TG/HDL levels were greater than 1.66. These results demonstrated that the serum TG/HDL levels were related to the risk for macrosomia in a dose-response manner.

## 4. Discussion

In this study, we found that the maternal serum TG/HDL levels in late gestation were independently and positively associated with the risk of macrosomia in nondiabetic women, which suggested that maintaining maternal serum lipids in an appropriate range is important in pregnant women to decrease the risk of macrosomia. To our knowledge, this study is the first to evaluate the association of TG/HDL in late gestation with the risk of macrosomia.

Maternal lipids are transferred across the placenta and play an important role in fetal development [15]. To support this function, increased lipidemia occurs throughout gestation. Mildly elevated TG and cholesterol are detected throughout pregnancy, with a more pronounced increase by the second and third trimesters [16]. Currently, there are no good markers to indicate dyslipidemia during pregnancy. No evidence currently supports interventions during pregnancy to reduce the risk of complications related to dyslipidemia [31]. The triglyceride-to-HDL cholesterol (TG/HDL) ratio has been reported to be closely related to insulin resistance in adults [32]. Several studies have found that the pregestational or early gestational TG/HDL was associated with GDM risk and adverse outcomes [29, 33, 34]. However, the relationship between late-gestational TG/HDL and macrosomia risk is unknown. The present study specifically explored the late-gestational TG/HDL ratio as a risk factor for macrosomia.

Although the primary goal of the aforementioned lipid alterations in pregnancy is nutritional supply to the fetus, these changes have been associated with several neonatal adverse outcomes [35], such as gestational hypertension, GDM, and preterm birth (PTB) [17–19]. Previous studies have also shown a significant correlation between maternal fasting TG levels and LDL levels in late pregnancy and neonatal birth weight [24]. The role of maternal hyperlipidemia in neonatal development is not yet fully understood. Special attention should be paid to the fact that the assessment and interpretation of laboratory parameters during pregnancy are complicated because of the changing levels of various hormones and several metabolic changes aimed at improving fetal access to nutrition and other factors [33].

Lipid and glucose metabolism are cross-linked and influence each other. Previous studies have mainly focused on the effect of abnormal elevation of lipids during pregnancy on adverse outcomes, especially in the GDM population [36, 37]. In our study, we used strict inclusion and exclusion criteria. We excluded all the women with GDM and DM history, and we further adjusted for the effect of FPG in our analysis. Furthermore, the maternal age and blood pressure levels were similar between the control and macrosomia groups in our population, so we could observe the direct effect of pregnancy dyslipidemia on macrosomia risk.

Our study also has important public health implications. Circulating plasma lipid patterns during normal pregnancy have been widely studied, and most studies have found that maternal dyslipidemia over a physiological range is a common phenomenon during pregnancy [38, 39]. Together with previous studies [17–19], our results provide new evidence for the adverse pregnancy outcomes of dyslipidemia during pregnancy. Until now, there have been no good markers to indicate pregnancy dyslipidemia. No evidence currently supports interventions during pregnancy to reduce the risk of complications related to dyslipidemia [31]. Our findings provide evidence to implement the recommendation of maintaining maternal lipid levels at the appropriate range during pregnancy and improve the lipid management plans during pregnancy.

Our study also had some limitations. First, the present study used a retrospective design, and selection and information bias, including selection bias such as Berkson's bias and exclusive bias, cannot be totally excluded. Some major confounders, including parity [8] and weight gain during pregnancy [40], were not adjusted because we lacked these data, which may be the reason that the goodness of fit of our multivariate linear regression model is not very high. Second, the sample size was still relatively small, so some possible confounders may not have been identified. Third, this was a single center study, and the ratio of pregnant women at high risk is relatively high in Shandong Provincial Hospital, which is a large-scale comprehensive three-A public hospital in Shandong province undertaking the tasks of medical care, teaching, scientific research, preventative care and guidance for local medical practices, and ranks first for comprehensive medical service in the province. The sample cannot be a representative of the population of China or the province. However, this was a case-control study, and we thought that the sample resources may not substantially conflict with the results. Further multicenter studies will be needed to overcome these limitations.

In conclusion, our results revealed that late-gestational TG/HDL is positively associated with an increased risk of macrosomia in nondiabetic women. Our findings demonstrated the importance of maintaining maternal lipid levels in appropriate ranges in the context of fetal overgrowth and primary prevention of macrosomia. However, further prospective investigations involving larger populations and basic research studies are necessary to fully evaluate their clinical value and the mechanisms involved. Further studies will have to be conducted using a prospective observational design to overcome the limitations of the present study.

## Figures and Tables

**Table 1:** Characteristics of the study population.

	Normal birth weight	Macrosomia	*P* value
Number	684	171	
Maternal characteristics			
Age (years)	30.44 ± 3.47	30.44 ± 3.48	1.000
Height (cm)	163.16 ± 4.85	164.84 ± 4.51	≤0.001
Weight (kg)	72.77 ± 8.98	80.24 ± 10.54	≤0.001
BMI (kg/m^2^)	27.33 ± 3.24	29.52 ± 3.76	≤0.001
SBP (mmHg)	118.98 ± 11.46	121.46 ± 11.37	0.012
DBP (mmHg)	76.11 ± 9.14	77.81 ± 8.71	0.025
Hypertension, *n* (%)	60 (8.77%)	16 (9.36%)	0.810
FPG (mmol/L)	4.35 ± 0.34	4.38 ± 0.37	0.266
UA (*μ*mol/L)	267.73 ± 61.71	275.90 ± 67.85	0.130
Creatine (*μ*mol/L)	51.90 ± 10.76	51.90 ± 9.36	0.999
AST (U/L)	17.68 ± 5.65	17.56 ± 5.66	0.428
ALT (U/L)	11.88 ± 8.21	10.72 ± 5.53	0.082
Neonatal characteristics			
Male sex (number, %)	347 (50.73%)	102 (59.65%)	0.037
Gestational age (weeks)	39.67 ± 1.08	39.99 ± 0.91	≤0.001
Birth length (cm)	49.33 ± 1.77	51.63 ± 1.45	≤0.001
Gravidity			0.431
1	367 (53.65%)	86 (50.29%)	
>1	317 (46.35%)	85 (49.71%)	
Delivery mode			0.015
Vaginal delivery	371 (54.24%)	75 (43.86%)	
Caesarean section	313 (45.76%)	96 (56.14%)	

All data are expressed as mean ± standard deviation or number (percentage). BMI, body mass index; SBP, systolic blood pressure; DBP, diastolic blood pressure; FPG, fasting plasma glucose; UA, uric acid; AST, aspartate transaminase; and ALT, alanine aminotransferase. There are 13, 18, 22, 2, and 4 missing data for the maternal height, maternal weight, maternal BMI, maternal UA, and delivery mode, respectively.

**Table 2:** Maternal lipid profile of the study population.

	Normal birth weight	Macrosomia	*P* value
Number	684	171	
TC (mmol/L)	6.73 ± 1.336	6.80 ± 1.35	0.562
HDL-C (mmol/L)	2.15 ± 0.56	1.80 ± 0.38	≤0.001
LDL-C (mmol/L)	3.46 ± 0.96	3.42 ± 0.98	0.108
TG (mmol/L)	2.57 (0.92)	3.64 (1.86)	≤0.001
TG/HDL	1.26 ± 0.43	2.27 ± 1.22	≤0.001
TG/LDL	0.80 ± 0.35	1.29 ± 0.93	≤0.001
LDL/HDL	1.68 ± 0.55	1.90 ± 0.60	≤0.001

All data are expressed as mean ± standard deviation or median (interquartile range). TC, total cholesterol; HDL-C, high-density lipoprotein cholesterol; LDL-C, low-density lipoprotein cholesterol; and TG, triglyceride.

**Table 3:** Correlations between neonatal body weight levels and clinical laboratory characteristics (*n* = 855).

	*r*	*P* value
Maternal age (years)	0.018	0.605
Gestational age (weeks)	0.247	≤0.001
SBP (mmHg)	0.088	0.010
DBP (mmHg)	0.003	0.923
BMI (kg/m^2^)	0.274	≤0.001
FPG (mmol/L)	0.048	0.165
Birth length (cm)	0.655	≤0.001
Male sex newborn	0.111	0.001
TC (mmol/L)	0.015	0.655
HDL-C (mmol/L)	−0.236	≤0.001
LDL-C (mmol/L)	−0.013	0.703
TG (mmol/L)	0.343	≤0.001
TG/HDL	0.422	≤0.001
LDL/HDL	0.173	≤0.001

**Table 4:** Multivariable linear regression analysis for the association between TG/HDL levels and neonatal body weights.

		Newborn body weight			
		Unstandardized beta	Standardized beta	95% CI of unstandardized beta	*P* value
TG/HDL (*n* = 855)	Unadjusted	252.76 ± 18.59	0.422	216.28–289.24	≤0.001
	Model 1	226.47 ± 19.06	0.376	189.06–263.87	≤0.001
	Model 2	216.73 ± 17.93	0.362	181.54–251.92	≤0.001

Data are standardized coefficients (beta) and significance values (*P* value). *Note.* Model 1, adjusted for BMI and FPG. Model 2, adjusted for maternal age, maternal height, gestational age, BMI, FPG, SBP, and neonatal sex. *P* < 0.05 was considered significant.

**Table 5:** Multivariate conditional odds ratio for the presence of macrosomia according to the categories of TG/HDL.

Quartile of TG/HDL	B	SE	OR	95% CI of OR	*P* value
Quartile 1 (≤1.02) (*n* = 214)			1		
Quartile 2 (1.02–1.32) (*n* = 214)	−0.120	0.413	0.887	0.395–1.991	0.771
Quartile 3 (1.33–1.65) (*n* = 214)	0.610	0.358	1.841	0.913–3.714	0.088
Quartile 3 (≥1.66) (*n* = 213)	2.582	0.324	13.229	7.015–24.950	≤0.001

CI, confidence interval; OR, odds ratio. Data are coefficient (B), corresponding SE, OR, 95% CI, and significance (*P* value). The multivariate conditional model was adjusted for maternal age, maternal height, gestational age, BMI, FPG, SBP, and neonatal sex.

## Data Availability

The data used to support the findings of this study are available from the corresponding author upon request.
